# Atorvastatin Decreases Renal Calcium Oxalate Stone Deposits by Enhancing Renal Osteopontin Expression in Hyperoxaluric Stone-Forming Rats Fed a High-Fat Diet

**DOI:** 10.3390/ijms23063048

**Published:** 2022-03-11

**Authors:** Chan Jung Liu, Yau Sheng Tsai, Ho Shiang Huang

**Affiliations:** 1 Department of Urology, National Cheng Kung University Hospital, College of Medicine, National Cheng Kung University, Tainan 704302, Taiwan; dragon2043@hotmail.com; 2 Department of Urology, College of Medicine, National Cheng Kung University, Tainan 70101, Taiwan; 3 Institute of Clinical Medicine, College of Medicine, National Cheng Kung University, Tainan 704302, Taiwan; yaustsai@mail.ncku.edu.tw; 4 Center for Clinical Medicine Research, National Cheng Kung University Hospital, College of Medicine, National Cheng Kung University, Tainan 704302, Taiwan

**Keywords:** atorvastatin, calcium oxalate, urolithiasis, atherosclerosis, hydroxyproline, hyperlipidemia

## Abstract

Calcium oxalate (CaOx) is the major constituent of kidney stones. Growing evidence shows a close connection between hyperlipidemia, cardiovascular disease (CVD), and the formation of kidney stones. Owing to their antioxidant properties, statins control hyperlipidemia and may ameliorate CaOx stone formation. The present study was designed to investigate the suppressive effects of statins on CaOx urolithiasis and their potential mechanism. We used rats fed a high-fat diet (HFD) to achieve hyperlipidemia (HL) and hydroxyproline (HP) water to establish a hyperoxaluric CaOx nephrolithiasis model; the animals were administered statins (A) for 28 days. The rats were divided into eight groups treated or not with A, i.e., Control, HP, HL, HL + HP. HL aggravated urinary calcium crystallization compared to the control. Due to increased expression of renal osteopontin (OPN), a key anti-lithic protein, and reduced free radical production, the calcium crystals in the urinary bladder increased as renal calcium deposition decreased. The levels of the ion activity product of CaOx (AP(CaOx)) decreased after statins administration, and AP(Calcium phosphate) (CaP) increased, which suggested the dominant calcium crystal composition changed from CaOx to CaP after statin administration. In conclusion, atorvastatin decreases renal CaOx stone deposits by restoring OPN expression in hyperoxaluric rats fed a HFD.

## 1. Introduction

Urinary tract stone disease, also known as urolithiasis, is one of the most common urologic diseases worldwide with increasing prevalence. Uroliths mostly consist of calcium oxalate (CaOx), ranging from 60% to 80%, and calcium phosphate (CaP) [1,2]. Accumulating evidence suggests that metabolic syndrome is closely associated with urolithiasis [3]. Several studies have also suggested that renal stone formers carry a higher risk of atherosclerosis than controls without stones [4]. Although numerous epidemiological studies have reported an association between metabolic syndrome and urolithiasis in recent years, the mechanism underlying this association is still unclear [5].

Dyslipidemia, the hallmark of metabolic syndrome, is linked to atherosclerosis. Oxidized low-density lipoprotein (oxLDL) is a key factor contributing to atherosclerosis [6]. Circulating oxLDL can stimulate foam cells in the arterial intima, where macrophages ingest massive amounts of oxLDL via scavenger receptors and lectin-like oxLDL receptor-1 (LOX-1) [7]. Macrophages can be stimulated by oxLDL to increase reactive oxygen species (ROS) production and enhance oxidative stress (OS), and these changes are closely related to CaOx kidney stone formation [8,9]. Some studies have reported that oxLDL may represent the connection between atherosclerosis, CVD risk, and urolithiasis, although these studies have yielded conflicting results [10].

Statins, or 3-hydroxy-3-methylglutaryl CoA (HMG-CoA) reductase inhibitors, are the mainstay of the primary prevention of dyslipidemia and CVD. Statin treatment also has antioxidative benefits aside from lowering systemic cholesterol levels, as reported by numerous studies [11,12]. Statins may have the potential to prevent urolithiasis considering the close connection between hyperlipidemia, OS, and urolithiasis. Observational and metabolic studies have demonstrated that patients taking statins have a reduced risk of urinary stones [13,14]. Our previous study revealed that statin administration could significantly alter urine citrate, UA, and pH in calcium kidney stone formers [15]. However, few studies have focused on the influence of statins on renal crystal retention using a hyperoxaluric stone-forming rat model [16]. Tsujihata et al., used hyperoxaluric rats to investigate the impact of atorvastatin treatment on urolithiasis and found a decrease in renal crystal retention after atorvastatin administration [17]. However, the precise mechanism of statin influence on CaOx kidney stones is not fully understood.

The clinical treatment of CaOx urolithiasis has so far been restricted to the empirical manipulation of urinary chemistry by potassium citrate, and no attempts have been made to design treatments for the underlying pathophysiology. As CaOx stones are the most common type of renal stones found in humans, we hypothesized in the current study that statin administration would alter high-fat-diet (HFD)-induced oxLDL-related renal injury and further reduce CaOx stone formation in hydroxyproline (HP)-induced hyperoxaluric rats. The present study also sought to determine whether the results from the study of statins in hyperoxaluric rats can be generalized to patients with predominant CaOx urolithiasis in a proof-of-concept interventional study.

## 2. Results

### 2.1. Serum and Urinary Biochemistry Change after Statins Use

Table 1 shows the data for the age-matched control and statin-treated groups. The rats in the HP and the A + HP groups had significantly lower body weights and food intake. The rats in the HL + HP and the A + HL + HP groups had significantly lesser urinary output than the controls (C). Renal function was significantly worse in rats fed HP, HL, or both, regardless of A use. Regarding the lipid profiles, total serum cholesterol and LDL levels were significantly increased after HFD induction (Table 2). Statin administration decreased cholesterol and LDL levels in HFD-fed rats compared to those not receiving statin. The changes in the atherogenic index were equal to those of cholesterol and LDL. The composition of electrolytes was generally similar in all groups, except for Pi, whose level was mildly higher in the HL + HP group.

Results of the 24 h urine analysis showed that urine Ca level was significantly lower in all rats fed a HFD than that in the control group (Table 2). The rats in the HP and HL + HP groups showed significantly lower levels of urine citrate than the control group. Urine oxalate (Ox) significantly increased in the HP and HL + HP groups compared to the control group.

### 2.2. Crystal Morphology and Deposition in the Kidney Interstitial Tissues and Urinary Sediments

To determine the effects of statins on calcium stone formation, we used the results from the 24 h urine analysis to calculate the urinary supersaturation with respect to CaOx and CaP, which was assessed using the index proposed by Tiselius et al., as the ion activity product of CaOx and CaP (AP(CaOx) and AP (CaP)) [18]. It was clearly observed that all the HP-fed rats had a significantly increased AP(CaOx) level compared with the control group. In contrast, rats in the HL and HL + HP groups had a significantly lower AP(CaP) level.

**Figure 1:**
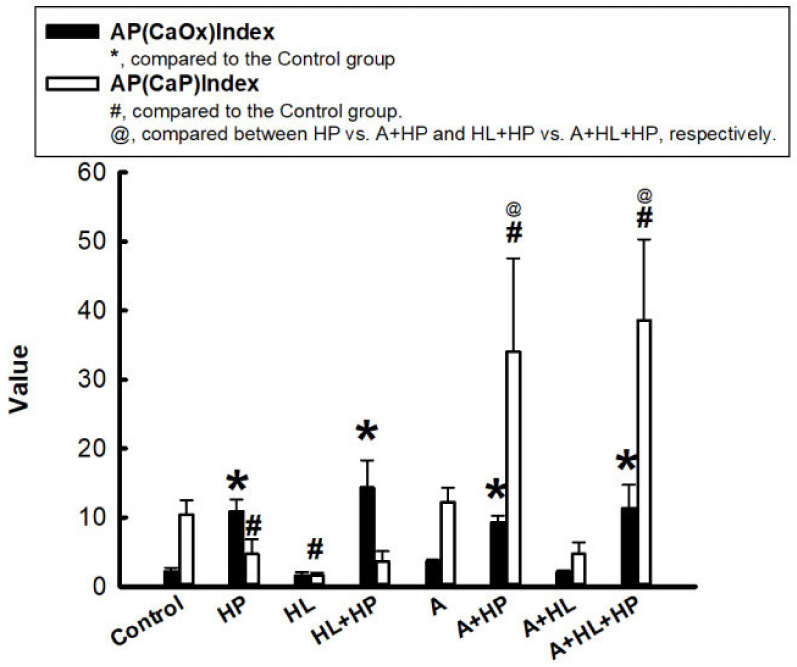
Ion activity product of calcium oxalate (AP(CaOx)) and calcium phosphate (AP(CaP)). The black bar represents AP(CaOx), and the white bar represents AP(CaP). * *p* < 0.05 versus AP(CaOx) of the control group; # *p* < 0.05 versus AP(CaP) of the control group; @ *p* < 0.05 between with and without A in each subgroup (e.g., N vs. A).

**Figure 2:**
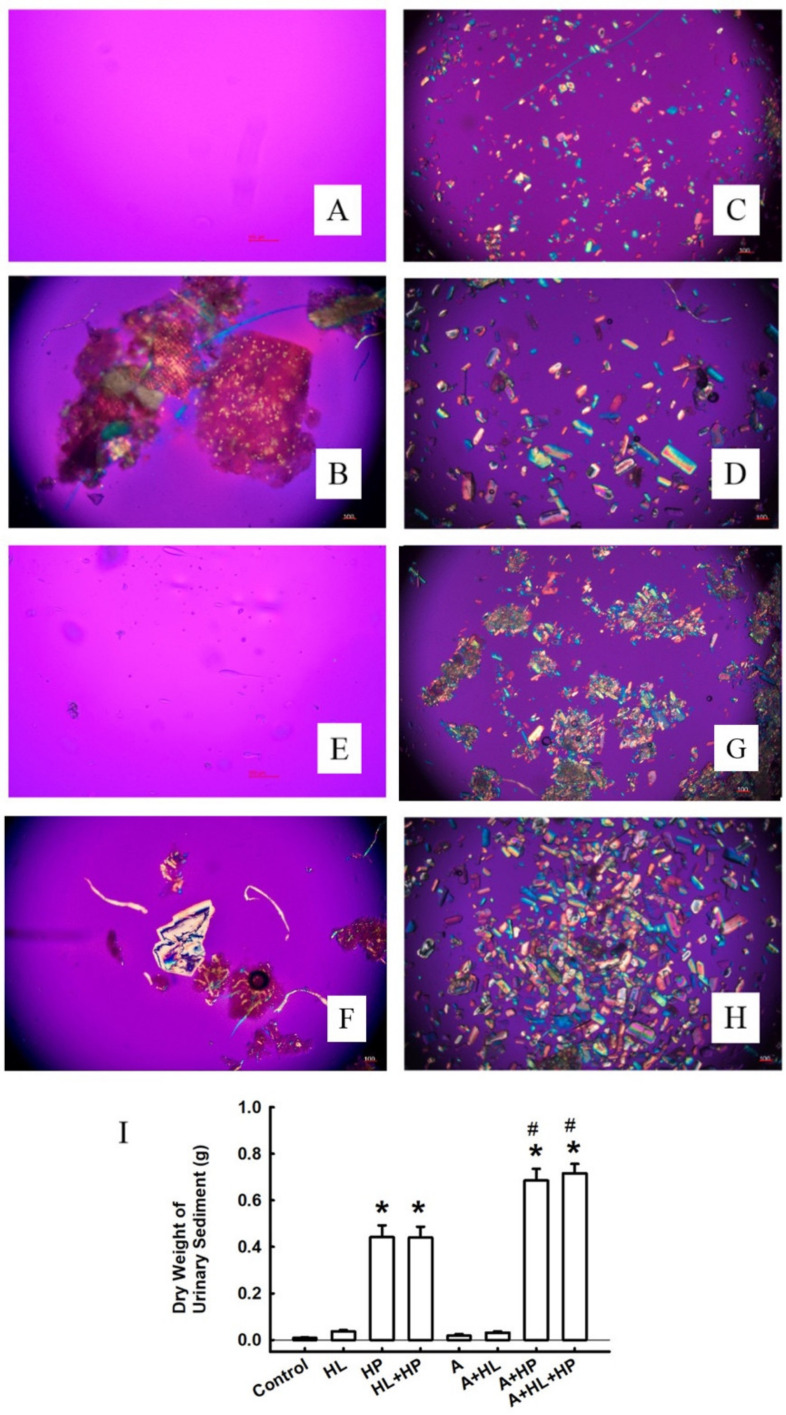
Representative micrographs of calcium crystals in urine sediments photographed using a polarized microscope. (**A**) Control. (**B**) Hyperlipidemia (HL). (**C**) Rats treated with 5% hydroxyproline (HP). (**D**) HL rats treated with 5% HP. (**E**) Controls treated with atorvastatin. (**F**) HL rats treated with atorvastatin. (**G**) Rats treated with 5% HP and atorvastatin. (**H**) HL rats treated with 5% HP and atorvastatin. All images above were demonstrated with original magnification 100×. (**I**) Statistical result for the dry weight of urine sediments. * *p* < 0.05 versus the control group; # *p* < 0.05 versus the controls treated with atorvastatin.

**Figure 3:**
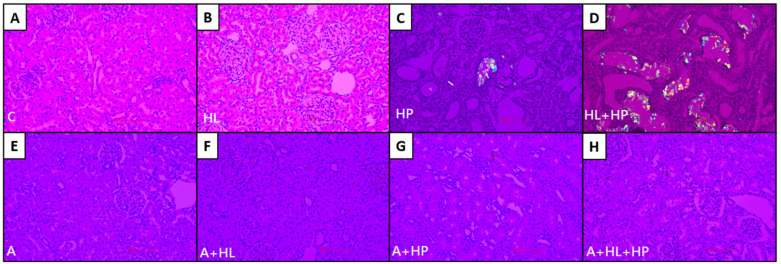
Representative micrographs of renal cortex and medulla photographed using a polarized microscope. (**A**) Control. (**B**) Hyperlipidemia (HL). (**C**) Rats treated with 5% hydroxyproline (HP). (**D**) HL rats treated with 5% HP. (**E**) Controls treated with atorvastatin. (**F**) HL rats treated with atorvastatin. (**G**) Rats treated with 5% HP and atorvastatin. (**H**) HL rats treated with 5% HP and atorvastatin. All images above were demonstrated with original magnification 200×.

However, AP(CaP) significantly increased in the A + HP and A + HL + HP groups compared to both the control group and the non-statin matched groups after statin administration (Figure 1). The treatment with statins did not significantly alter AP(CaOx). Crystalluria revealed the presence of calcium crystals in all groups other than the control group (Figure 2). The mean crystalline volume was significantly higher in HP-fed rats. Interestingly, statin administration significantly increased the mean crystalline volume especially in HP-fed and statin-treated rats. The appearance of calcium crystals in urinary sediments significantly changed after statin use. Calcium crystals before statin use are reported to be CaOx [19]. Conversely, statin administration converts calcium crystals into CaP, which typically appears magenta in the background and are characterized by orange and blue quadrants [20]. No CaOx crystals were observed in the renal sections of the control and HL groups (Figure 3A,B), but the crystals were detectable in both the renal cortex and the medulla in the HP and HL + HP groups (Figure 3C,D). The abundance of CaOx crystal deposition was significantly higher in the HL+ HP group than in the HP group (Gr. II–III vs Gr. I–II). In contrast, the deposition of calcium crystals notably decreased after statin use and was more prominent in the kidney interstitial space (Figure 3G,H).

### 2.3. Detection of Cell Death In Situ and Its Relationship with Macrophages

**Figure 4:**
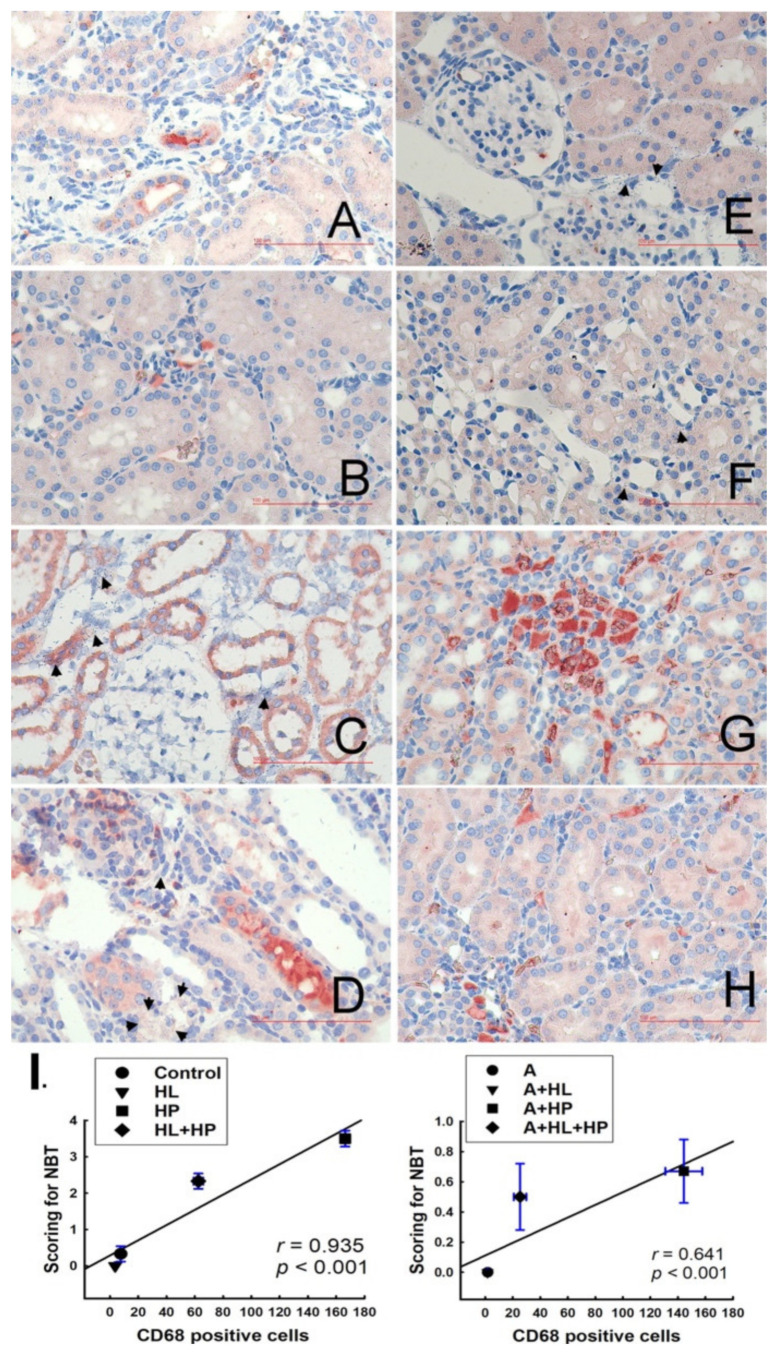
Representative micrographs of renal sections double staining for in situ superoxide formation (indicated by NBT, blue formazan particles, black arrowheads) and CD68 (monocyte and macrophage marker) immunostaining. Left panel: **A**–**D**: Non-A (Atorvastatin) experimental groups; Right panel: **E**–**H**: A-treated experimental groups. (**A**) Control group: only a few orange-red stained CD68-positive cells with NBT score of 0+. (**B**) Hyperlipidemia (HL) group: 3–5 CD68-positive cells in the interstitium, with NBT score of 0+. (**C**) Rats treated with 5% hydroxyproline (HP): CD68-positive cells located in dilated renal tubule cells and widened interstitium, with NBT of 3+-4+. (**D**) HL+HP group: many CD68-positive cells located in the interstitium with NBT score of 2+-3+ (**E**) A group: 1–5 CD68-positive cells in the glomerulus, with NBT score of 0+. (**F**) A+HL group: 1–5 CD68-positive cells, with NBT score of 0+. (**G**) A+HP group: many dark-stained CD68-positive cells in the interstitium, with NBT score of 0+-1+. (**H**) A+HL+HP group: 15–30 CD68-positive cells, with NBT score of 1+. All images above were demonstrated with original magnification 400×. (**I**) Relationship between changes in NBT scoring and CD68-positive cells in immunostained kidney samples; positive correlation is shown, with r = 0.935, *p* < 0.001 in the non-A treated groups and r = 0.641, *p* < 0.001 in the A-treated group.

NBT staining is an effective method to detect superoxide dismutase (SOD), which can be observed as NBT-positive (purple-blue) or NBT-negative (yellow-tan) under bright-field microscopy. No blue formazan particles were detected in the control and HL groups; therefore, we recorded the NBT score as 0+, according to a previous study [19]. However, blue formazan particles densely surrounded the renal tubules in the HP group (score 3+-4+) (Figure 4C). Blue formazan particles were also detected in the kidney interstitial space (score 2+-3+) in the HL + HP group (Figure 4D). Significant renal tubule dilation and tubular cell apoptosis were also observed in the two aforementioned groups. Additionally, NBT expression significantly decreased in the two groups compared with the matched groups after statin administration (Figure 4E–H). We performed double immunohistochemical staining with NBT and anti-CD68 antibodies to determine the association between SOD formation and macrophages. Positive CD68 expression was detected as orange-red and was mainly localized in the kidney interstitial spaces in the control and HL groups (Figure 4A,B). CD68 expression was significantly increased in the HP and HL + HP groups and was mainly localized in renal tubule cells (Figure 4C,D). CD68 expression also increased in the matched HP and HL + HP groups after statin administration. A strong and significant correlation between CD68-positive cells and NBT score in all groups was found (Figure 4I). This result indicates that macrophage recruitment may be directly involved in the production of SOD during renal stone formation.

### 2.4. Impacts of Statin on the Expression of Lectin-like Ox-LDL Receptor-1 (LOX-1), Antilithic Proteins, and Tubule Injury Markers in the Kidney

**Figure 5:**
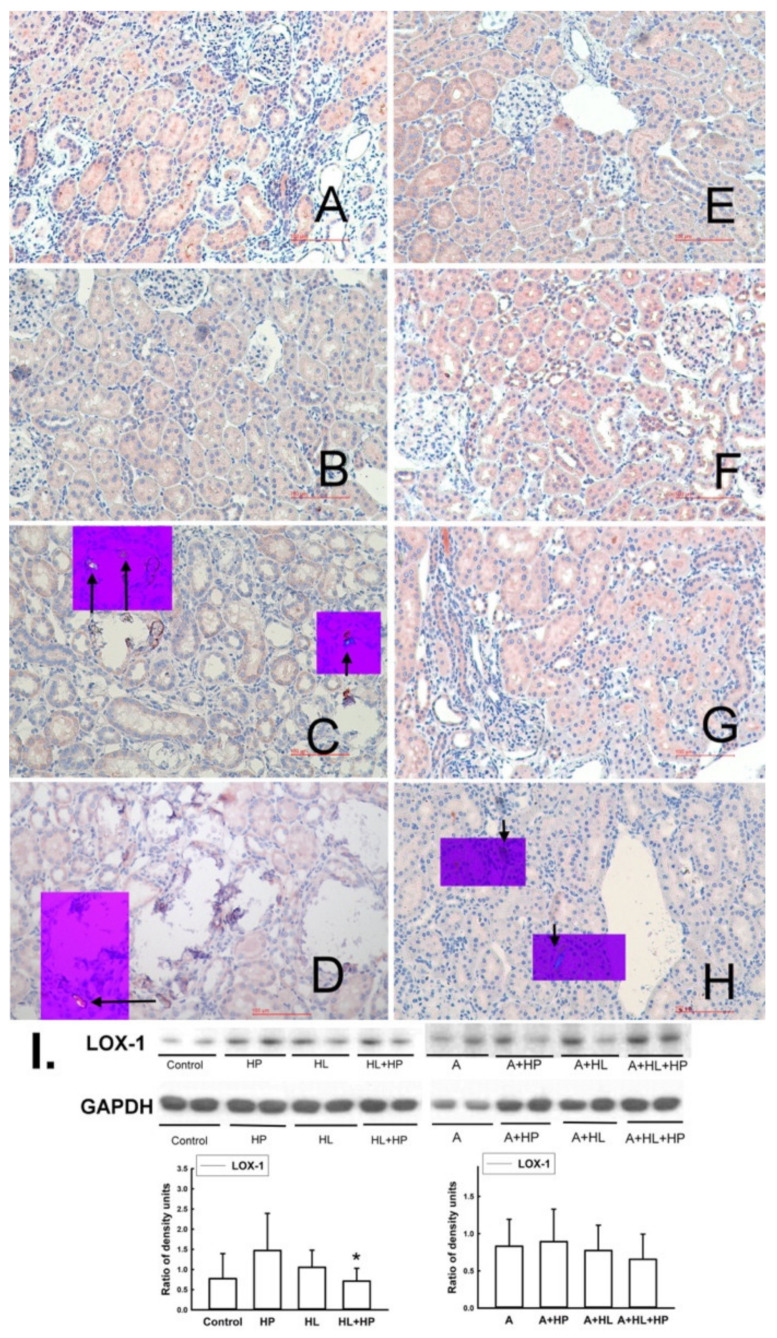
Representative micrographs of renal sections stained for LOX-1 (oxidized LDL receptor), Reduced from × 200. The small purple box shows the same renal section which was examined by polarized microphotography (Reduced from × 200) to observe CaOx or CaP deposition (indicated by black arrows) in the kidney. Left panel: **A**–**D**: Non-A (Atorvastatin) experimental groups: (**A**) Control group. (**B**) Hyperlipidemia (HL). (**C**) Rats treated with 5% hydroxyproline (HP). (**D**) HL rats treated with 5% HP: LOX-1 level decreased significantly compared to the levels in the control group (*p* = 0.034) and the HP group (*p* = 0.026). Right panel: **E**–**H**: A-treated experimental groups: (**E**) A group. (**F**) A + HP group. (**G**) A + HL group. (**H**) A + HL + HP group. All images above were demonstrated with original magnification 400×. (**I**) The representative blots shown were obtained from two different rat kidneys. The histogram shows the relative density of the protein of interest with respect to GAPDH density for six animals in each groups. * *p* < 0.05 when compared to the age-matched corresponding HP group.

The expression of LOX-1, a specific scavenger receptor of oxLDL, decreased significantly only in the HL + HP group (*p* = 0.029) compared to the HP groups (Figure 5). However, there was no significant change in the expression of LOX-1 after administration of statins.

**Figure 6:**
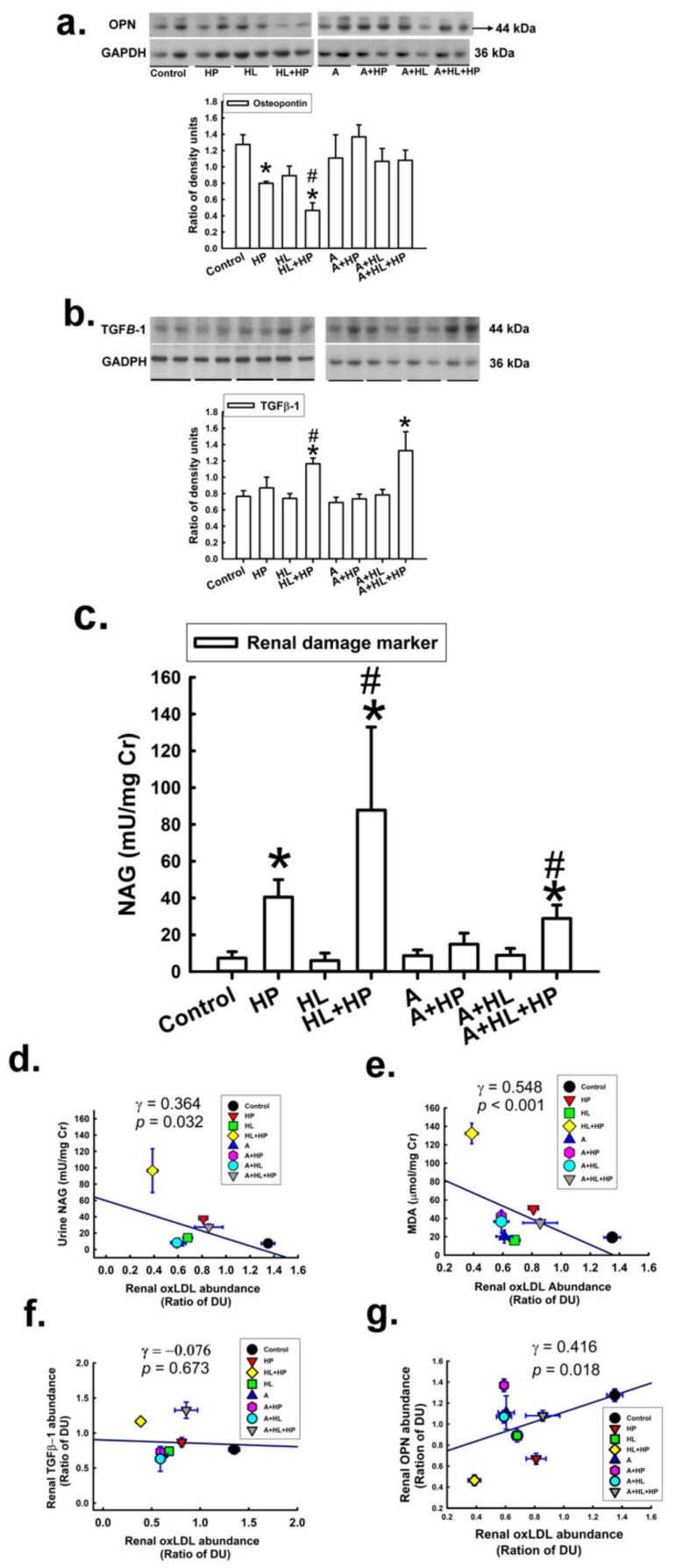
Impact of atorvastatin on the expression of (**a**) the antilithic molecule osteopontin (OPN), * versus control; # versus HL, (**b**) transforming growth factor (TGFβ-1, is a critical mediator of organ fibrosis), * versus control; # versus HL, (**c**) renal tubular damage markers (N-acetyl-β-glucosaminidase, NAG), * versus control; # versus HL. (**d**–**g**) show the correlationship between kidney oxLDL level (relative DU) and urine NAG, urine MDA, and renal TGFβ-1 and OPN. Kidney oxLDL showed a positive correlation with urine MDA (γ = 0.548, *p* < 0.001) and renal OPN (γ = 0.416, *p* = 0.018) and a negative correlation with urinary NAG levels (γ = 0.364, *p* = 0.032).

Osteopontin (OPN) is a critical inhibitor of kidney stone formation, and its expression in the kidney was reported to be decreased in patients with nephrolithiasis and in an animal model of nephrolithiasis [21]. Consistent with the results of the previous study, we found that the expression of OPN in the kidneys was significantly decreased in the HP and HL + HP groups in the current study (Figure 6a). Statin administration restored OPN expression. Additionally, the abundance of OPN was significantly related to the abundance of renal oxLDL. To evaluate the impact of statins on kidney injury, we performed transforming growth factor (TGF) immunohistochemistry (IHC) staining and used urinary N-acetyl-β-d-glucosaminidase (NAG) and malondialdehyde (MDA) as markers (Figure 6b,c). The expression of TGF and urinary NAG was significantly increased in the HL + HP group compared with the control group, and this pattern was also noted in the statin-treated groups. These results indicated that the combination of HL and HP caused more kidney injury than HP or HL alone. The levels of urinary NAG and MDA were significantly and negatively correlated with renal oxLDL expression, but positively correlated with renal OPN expression (Figure 6d–g).

## 3. Discussion

Due to the strong association between urolithiasis and atherosclerotic vascular disease, studies on the therapeutic effects of statins on urolithiasis can be traced back to more than 10 years [17]. Atorvastatin is reported to decrease kidney injury and oxidative stress in hyperoxaluric rats and inhibit renal crystal retention [17]. Studies on two large cohorts also found that statin administration was associated with a decreased risk of urolithiasis [13,14]. These results suggest the possible protective potential of statins against urolithiasis, but the exact mechanism of protection remains unclear. All related studies failed to specify the composition of stones in their cohorts, which limits the accuracy of their findings, although they presented statistically significant results. In the present study, we used rats with HP-induced hyperoxaluria urolithiasis to investigate the effects of statins on CaOx stone formation. Our results are related to previous human-based studies, which showed that statins could reduce renal CaOx deposits. To dissect the mechanism underlying these observations, we analyzed bladder urinary sediments and kidney specimens from hyperoxaluric rats and found that statin administration decreased renal calcium crystal deposits and increased urinary crystal expulsion. A possible explanation for this inverse trend may be related to the effects of statins on anti-lithic proteins, free radical production, and adhesion molecules. Firstly, several anti-lithic proteins have been identified, and OPN is one of the major inhibitors of calcium crystal deposition [21]. We found that atorvastatin administration restored the expression of OPN in the kidney. Secondly, free-radical injury is known to promote CaOx stone formation [19]. In the present study, in situ detection of superoxide anions by NBT staining and costaining with anti-CD68 antibodies showed that the number of formazan particles increased in the HL+HP group and then decreased in all statin-treated groups, compared with the controls. This finding suggested that atorvastatin could ameliorate renal free-radical injury, which might contribute to the decrease in renal crystal deposits. Finally, previous studies found that statins inhibit the expression of adhesion molecules such as vascular cell adhesion molecule-1 (VCAM-1) and intercellular cell adhesion molecule-1 (ICAM-1) in vascular endothelial cells, preventing atherosclerosis progression [22]. Statins can also ameliorate renal ischemia reperfusion injury by suppressing tumor necrosis factor-α (TNF-α)-induced VCAM-1 expression [23]. Our previous studies showed that the serum levels of VCAM-1 and ICAM-1 were elevated in patients with urolithiasis [4,24]. Additionally, a previous study investigating the differential roles of M1/M2 macrophages in the development of renal CaOx stones found that M1 macrophages induced the expression of adhesion-related genes and facilitated renal crystal formation [25]. All these results support the hypothesis that decreased expression of adhesion molecules could lead to the inhibition of renal crystal deposition. Hence, our findings can relate the effect of statins on adhesion molecules in the kidney, which contribute to increased calcium crystal expulsion.

A significant finding of this study is the conversion of CaOx to CaP. Before discussing the possible underlying mechanism of this conversion, knowledge on the physico–chemical mechanisms responsible for crystal growth and dissolution should be presented [26]. Calcium oxalate renal stones contain three phases, i.e., monohydrate (COM), dihydrate (COD), and trihydrate (COT). Of these, COM is the most stable and the final stage of CaOx, which is the most frequently observed type of kidney stones. CaP renal stones consist of at least four possible phases: brushite, nanocrystalline octacalcium phosphate (OCP), hydroxyapatite (HAP), and amorphous calcium phosphate (ACP). Of these, the brushite phase has the highest solubility and the least stability. Brushite sequentially transforms into the other CaP phases with greater stability until the formation of HAP, the most thermodynamically stable phase. There is a theoretical thermodynamic driving force that converts brushite into COM in mixed oxalate/phosphate systems [26]. This conversion is controlled by urinary pH, calcium, and inhibitory macromolecules. The contra-thermodynamic transformation of COM to CaP occurs when the supersaturation of HAP increases considerably with a slight pH increase, but COM supersaturation remains almost the same. Undissolved HAP consumes urinary calcium and limits the formation of COM. Additionally, previous studies found that urinary macromolecules, especially OPN, inhibit renal COM formation and promote the contra-thermodynamic transformation to CaP [27]. An increased citrate supply and decreased urinary uric acid levels are associated with the conversion of CaOx to CaP [28,29]. Besides, the different chemical characteristics of oxalic acid and citrate, including solubility and dissociation constants, could be the possible cause of the conversion of CaOx to CaP. Urinary oxalic acid and citrate metabolism interact, though the mechanism is still uncertain, and can be influenced by several factors, including hyperuricemia, urinary upper metastable limit osmolality, and the microbiota [30,31,32]. Although we could not demonstrate an increase in urinary pH in the present study, we found that renal OPN expression significantly increased after statin treatment. Further studies are still warranted to identify the possible mechanism.

Accumulating evidence suggests that patients with urolithiasis have a higher risk of hyperlipidemia and atherosclerosis [33]. One study suggested that oxLDL may be the connection between urolithiasis and atherosclerosis [10]. The majority of oxLDL accumulates in the liver, and less than 2% of the total oxLDL is taken up by the kidneys [34]. Previous studies revealed oxLDL overexpression in the kidneys of patients with CKD or end-stage renal disease [35]. The possible detrimental link between oxLDL and the kidney is based on the concept of the “lipid nephrotoxicity”. Hyperlipidemia resulting from the compensatory hepatic synthesis of lipoproteins in response to urinary albumin leak can aggravate glomerular sclerosis and interstitial fibrosis [36]. Recently, an increasing number of studies have focused on oxLDL in relation to lipid nephrotoxicity. OxLDL can cause damage in renal mesangial cells, endothelial cells, and podocytes [37]. An in vivo study using human glomerular mesangial cells reported oxLDL uptake via LOX-1 accompanied by increased ROS production, further enhancing oxLDL endocytosis [38]. However, increased severity of renal injury after HFD intake but decreased expression of renal LOX-1 were found in the current study, which is in contrast to the findings in previous reports. More studies are needed to confirm our findings.

## 4. Materials and Methods

### 4.1. Experimental Induction of Renal CaOx Crystals and Atorvastatin Treatment

A total of 48 male Wistar rats (BioLASCO, Taipei, Taiwan) weighing 200–220 g were divided into eight groups in equal numbers. The HP groups were given 5% HP in distilled drinking water to induce hyperoxaluria as described previously [39]. The HL groups were fed a HFD to induce hyperlipidemia, as described previously [40]. The duration of hyperlipidemia induction was 8 d, and each rat was weighed, recorded, and randomized into two subgroups (HP and non-HP) for further experiments. Atorvastatin (A) was administered at a dose of 20 mg/kg/day by oral gavage. The eight groups were as follows: Control, HP, HL, HL + HP, A, A + HP, A + HL, and A + HL + HP. The non-HP groups, containing control, HL, A, and A + HL, were given distilled water to drink. The non-HL groups, including control, HP, A, A + HP, had free access to ground rat chow containing 50.83 g (per kg dry matter) of crude fat, but no cholesterol (C 1000; Altromin, Lage, Germany). The animals were placed in metabolic cages 3 d before euthanization and for acclimatization before the collection of 24 h urine samples and basic data, including water and food intake, weight gain, and blood samples, for further analysis. Urine calcium (Ca), oxalate (Ox), citrate (Cit), uric acid (UA), magnesium (Mg), phosphate (P), and pH were measured. Urine supersaturation with respect to CaOx and CaP was calculated using the index proposed by Tiselius et al. [18]. The animal care and experimental protocols followed the Guide for the Care and Use of Laboratory Animals and were reviewed and approved by the Institutional Animal Care and Use Committee.

### 4.2. Immunohistochemistry and Nitro Blue Tetrazolium Staining

The rat kidneys were perfused using a transcardiac method with cold phosphate-buffered saline (0.1 M sodium phosphate buffer (PBS), pH 7.4) as described previously [39] and post-fixed overnight in 4% formalin solution containing 5% zinc sulfate, and 5 mm-thick sections were prepared. The sections were stained with hematoxylin and eosin. Deparaffinized sections were incubated with 3% non-fat milk to eliminate non-specific binding and then with an endogenous peroxidase inhibitor (OriGene Technologies Inc., Beijing, China), according to the manufacturer’s protocol, with both incubations performed at room temperature for 30 min. The sections were then incubated with primary anti-LOX-1 antibody (1:200 dilution, Abcam, Cambridge, MA, USA), or primary polyclonal OPN antibody (1:200 dilution, Lucerna-Chem, Luzern, Switzerland) for 60 min at room temperature, followed by a 15 min wash in PBS. The sections were then incubated with goat anti-rabbit IgG (1:500 dilution) for 60 min at room temperature. A nitro blue tetrazolium (NBT, Cayman Chemicals, Michigan, USA) perfusion test was performed to localize the de novo ROS production in the kidney [19]. The details about the localization of superoxide generation (blue formazan particles) in the kidney are presented in Supplementary Materials.

### 4.3. Detection of Calcium Crystals in Renal Sections and Urine Sediment

The crystal deposits in the kidney sections were clearly visible under a NIKON Ci-L upright polarized-light microscope. Each kidney was scored semi-quantitatively as one of four grades (0, I, II, III), ranging from “no” (0) to “massive” (III) crystal deposit, as described previously [19]. Debris in the 24 h urine samples and bladder urine collected from the rats at the time of death were gathered to determine the presence of calcium crystals by polarized microscopy after placing the samples in an oven for 48 h. The sediments were weighed until their dry weight was stable, as described previously [19].

### 4.4. Determinants of Superoxide Formation and Associations with Monocytes/Macrophages

The rats received transcardiac perfusion with PBS, followed by perfusion with 40 mg of NBT (1 mg/mL) by the same route, as described previously, after being anesthetized. After right kidney nephrectomy, the left kidney was perfused with a 1 mg/mL solution of NBT at 4 °C at a rate of 2 mL/min for 20 min. The perfused kidney was then fixed and embedded in paraffin, and 5 mm sections were prepared.

The kidney specimens were deparaffinized directly for observation under a light microscope to observe the localized superoxide generation (blue formazan particles) in the kidneys and to determine its correlation with infiltrated monocytes/macrophages. The localization of superoxide generation was indicated by the deposition of blue formazan particles and scored (at × 400 magnification) as 0+ to 4+, where 0+ equaled 0–100 formazan particles per field, 1+ indicated 101–200 particles, 2+ indicated 201–300 particles, 3+ indicated 301–400 particles, and 4+ indicated 401–500 particles. The CD68 antigen, a 110 kDa type I transmembrane glycoprotein which is specific to monocytes and macrophages, (Serotec, Oxford, United Kingdom), was diluted 1:1000. CD68-positive cells were readily detectable in the kidney tissue by light microscopy, and the number of CD68-positive cells was counted under × 400 magnification.

### 4.5. Western Blot Analysis

The kidney specimens were placed in RIPA buffer containing a protease inhibitor cocktail and homogenized. The tissue lysate was centrifuged, and the protein concentration of the resulting supernatant was determined using a protein assay kit (Bio-Rad Laboratories, Hercules, CA, USA). Samples containing 10 μg of total protein were mixed with SDS loading buffer, boiled, electrophoresed in 10% SDS-PAGE gels, and then transferred onto PVDF membranes. The membranes were then blocked with blocking buffer for 1 h at room temperature and incubated overnight at 4 °C with anti-LOX-1 antibodies. After washing, the membranes were incubated with horseradish peroxidase-conjugated secondary antibodies. Immunoreactive protein detection was performed using an enhanced chemiluminescence detection system (PerkinElmer, Waltham, MA, USA).

### 4.6. Statistical Analysis

Numerical data are presented as the mean ± SD. Paired *t* tests were performed to compare the treatments. All other results were analyzed using unpaired *t* tests. The correlation between the groups was assessed using linear regression analysis. Statistical significance was set at *p* < 0.05.

## 5. Conclusions

We found that hyperlipidemic rats formed more CaOx stones after hydroxyproline intake compared to rats fed a normal chow. The administration of statins led to a significant reduction in stone formation. This reduction may be related to an increase in urine calcium crystal expulsion. The possible mechanism underlying these findings probably involves the increased expression of OPN in the kidney. Additionally, we showed statin administration facilitated the conversion of CaOx to CaP; to the best of our knowledge, this finding has not been reported yet. Our study provides new mechanistic insights into statins regulation of CaOx urolithiasis and could aid to establish statin as a novel therapeutic agent for the treatment of CaOx stone formation.

## Figures and Tables

**Table 1:** Summary of body data, urinary chemistry, and crystal deposition.

	N	HL	HP	HL + HP	A	A + HL	A + HP	A + HL + HP
BW (g)	408.2 ± 7.9	417.0 ±20.4	328.8 ± 21.4 ^a^	365.1 ± 12.1	395.5 ± 8.1	422.3 ± 10.7	334.3 ± 14.4 ^a,b^	407.3 ± 9.4
Food intake (g/24 h)	29.6 ± 1.0	28.2 ± 1.6	21.0 ± 1.4 ^a^	21.4 ± 3.0 ^a^	30.6 ± 0.5	29.9 ± 1.3	22.8 ± 2.4 ^a,b^	23.2 ± 2.6 ^a,b^
Water intake (mL/24 h)	50.6 ± 6.2	51.7 ± 8.0	36.8 ± 4.6 ^a^	35.0 ± 6.8 ^a^	51.1 ± 5.6	46.7 ± 2.7	36.1 ± 3.2 ^a,b^	31.7 ± 2.6 ^a,b^
UO (mL/24 h)	41.0 ± 5.3	27.1 ± 8.2	23.5 ± 3.7	15.6 ± 2.5 ^a^	25.4 ± 3.7	12.8 ± 0.7	20.2 ± 2.1	11.1 ± 0.9 ^a,b^
Ccr (mL/min)	5.2 ± 1.0	3.0 ± 0.6 ^a^	2.6 ± 0.5 ^a^	2.5 ± 0.8 ^a^	4.6 ± 0.9	1.8 ± 0.4 ^a,b^	2.1 ± 0.1 ^a,b^	1.6 ± 0.3 ^a,b^
Urine pH	7.6 ± 0.1	6.7 ± 0.1	6.9 ± 0.4	6.8 ± 0.6	7.2 ± 0.1	6.8 ± 0.1	6.8 ± 0.0	6.6 ± 0.4
Kidney Crystal deposit (score)	0	0	I–II	II–III	0	0	0–I	0–I

**Abbreviations:** N, normal; HL, hyperlipidemia; HP, hydroxyproline; A, atorvastatin; BW, body weight; UO, urine output; Ccr, creatinine clearance rate. ^a^ *p* < 0.05 vs. n alone. ^b^ *p* < 0.05 vs. A alone.

**Table 2:** Serum biochemistry and 24 h urinary profiles of each group.

	N	HL	HP	HL + HP	A	A + HL	A + HP	A + HL + HP
Serum								
TG (mg/dL)	101.5 ± 6.3	78.6 ± 9.8 ^a^	99.5 ± 2.7	87.3 ± 6.1	86.3 ± 13.8	86.7 ± 10.1	80.0 ± 12.1 ^c^	94.0 ± 28.0
Cholesterol (mg/dL)	63.3 ± 6.0	728.7 ± 104.9 ^a^	67.0 ± 2.9	361.0 ± 60.7 ^a^	68.0 ± 2.5	602.0 ± 84.0 ^a,b,c^	47.0 ± 1.6 ^c^	488.5 ± 66.8 ^a,b^
HDL (mg/dL)	21.8 ± 2.0	6.3 ± 2.2 ^a^	20.3 ± 1.7	10.7 ± 2.3 ^a^	22.8 ± 1.3	5.3 ± 1.8 ^a,b^	18.7 ± 0.7	8.3 ± 2.0 ^a,b^
LDL (mg/dL)	7.0 ± 0.4	154.1 ± 28.9 ^a^	7.8 ± 0.8	56.5 ± 15.8 ^a^	7.5 ± 0.7	119.2 ± 21.2 ^a,b,c^	6.7 ± 0.4	90.8 ± 17.6 ^a,b,c^
Atherogenic Index	2.0 ± 0.2	144.5 ± 22.0 ^a^	2.1 ± 0.2	58.7 ± 23.1 ^a^	1.7 ± 0.1	193.4 ± 70.8 ^a,b^	1.4 ± 0.1	88.4 ± 23.3 ^a,b,c^
Na (meq/L)	141.5 ± 0.7	136.2 ± 1.7	138.7 ± 1.4	136.7 ± 2.2	140.3 ± 1.3	135.2 ± 2.2	137.7 ± 2.3	136.0 ± 2.4
K (meq/L)	5.5 ± 0.3	5.7 ± 0.2	5.5 ± 0.3	5.5 ± 0.2	5.6 ± 0.3	5.5 ± 0.1	5.8 ± 0.5	5.5 ± 0.2
Ca (mg/dL)	10.2 ± 0.2	10.5 ± 0.2	9.6 ± 0.2	10.3 ± 0.1	10.0 ± 0.2	10.6 ± 0.2	9.7 ± 0.2	10.4 ± 0.2
P (mg/dL)	9.0 ± 0.1	7.7 ± 0.2	8.1 ± 0.2	7.4 ± 0.2	8.1 ± 0.2	7.5 ± 0.4	6.9 ± 0.5	7.5 ± 0.2
Urine								
Na (mmol/24 h)	2.9 ± 0.5	3.1 ± 0.4	1.9 ± 0.5	1.8 ± 0.3	3.1 ± 0.5	2.7 ± 0.4	1.6 ± 0.2	1.7 ± 0.5
K (mmol/24 h)	6.6 ± 0.6	2.9 ± 0.4 ^a^	4.1 ± 1.2	2.1 ± 0.5 ^a^	6.4 ± 1.1	2.4 ± 0.3 ^a,b^	3.3 ± 0.5	1.7 ± 0.4 ^a,b^
Ca (mg/24 h)	0.24 ± 0.11	0.05 ± 0.01 ^a^	0.33 ± 0.26	0.05 ± 0.02 ^a^	0.29 ± 0.06	0.07 ± 0.01 ^a,b^	0.12 ± 0.04 ^a,b^	0.06 ± 0.01 ^a,b^
P (mg/24 h)	40.0 ± 12.8	46.3 ± 6.4	35.5 ± 6.0	47.6 ± 17.3	24.3 ± 4.6 ^a,c^	35.1 ± 5.7	17.6 ± 2.1 ^a,c^	30.0 ± 6.2 ^a,c^
Mg (μmol/24 h)	35.4 ± 9.4	15.9 ± 5.4 ^a^	71.1 ± 26.9 ^a^	14.9 ± 5.3 ^a^	80.1 ± 31.1 ^a,c^	8.3 ± 1.4 ^a,b,c^	60.1 ± 27.2 ^a,b^	10.6 ± 2.6 ^a,b^
Protein (mg/24 h)	25.0 ± 2.7	22.0 ± 8.3	12.8 ± 3.8 ^a^	22.7 ± 5.2	24.4 ± 7.4	11.4 ± 2.5 ^a,b,c^	7.3 ± 1.2 ^a,b,c^	11.7 ± 3.3 ^a,b,c^
MDA (μmol/mg Cr)	19.2 ± 2.0	26.6 ± 11.3	52.2 ± 2.0 ^a^	141.3 ± 15.1 ^a^	24.1 ± 6.3	29.2 ± 9.6	48.6 ± 4.7 ^a,b^	62.3 ± 21.0 ^a,b,c^
Cit (mg/mg Cr)	2.4 ± 0.7	4.6 ± 2.0 ^a^	1.0 ± 0.2 ^a^	2.0 ± 0.5 ^a^	3.7 ± 0.7 ^a,c^	5.8 ± 2.7 ^a,b^	4.7 ± 1.0 ^a,c^	5.8 ± 2.6 ^a,b,c^
Ox (mg/24 h)	4.0 ± 0.7	4.6 ± 0.9	12.1 ± 1.6 ^a^	7.3 ± 1.7 ^a^	10.3 ± 2.0 ^a,c^	5.2 ± 1.2 ^b^	10.4 ± 1.2 ^a^	4.9 ± 0.8 ^b,c^

**Abbreviations:** N, normal; HL, hyperlipidemia; HP, hydroxyproline; A, atorvastatin; TG, triglyceride; HDL, high-density lipoprotein; LDL, low-density lipoprotein; MDA, malondialdehyde; Cit, citrate; Ox, oxalate; atherogenic index = (total cholesterol–HDL cholesterol)/(HDL cholesterol). ^a^ *p* < 0.05 vs. *n* alone. ^b^ *p* < 0.05 vs. A alone. ^c^ *p* < 0.05, comparison between with and without A in each subgroup (e.g., N vs. A).

## Data Availability

Not applicable.

## Supplementary Materials

The following are available online at https://www.mdpi.com/article/10.3390/ijms23063048/s1.
