# Supplementary material for: Atorvastatin Decreases Renal Calcium Oxalate Stone Deposits by Enhancing Renal Osteopontin Expression in Hyperoxaluric Stone-Forming Rats Fed a High-Fat Diet
Source: Int J Mol Sci. 2022 Mar 11;23(6):3048. doi: 10.3390/ijms23063048 (PMC8954580; doi:10.3390/ijms23063048)
Supplement: Supplementary file 1 [file ijms-23-03048-s001.zip › ijms-1618741-supplementary.pdf]

Determinants of superoxide formation and the associations with monocytes/macrophages

The rats received transcardiac perfusion with PBS followed by perfusion with 40 mg of NBT (1 mg/mL) by the same route as described previously after being anesthetized.[23] After right kidney nephrectomy, the left kidney was perfused with a 1 mg/mL solution of NBT at 4 °C at a rate of 2 mL/min for 20 min. The perfused kidney was then fixed and embedded in paraffin, and 5 mm sections were prepared.

The kidney specimens were deparaffinized directly for observation under a light microscope to observe localized superoxide generation (blue formazan particles) in the kidneys and to determine their correlation with infiltrated monocytes/macrophages. The localization of superoxide generation was indicated by the deposition of blue formazan particles and scored (at × 400 magnification) as 0+ to 4+, where 0+ equaled 0–100 formazan particles per field, and that at 1+ was 101–200, 2+ was 201–300, 3+ was 301–400, and 4+ was 401–500 particles. The CD68 antigen, a 110 kDa type I transmembrane glycoprotein which is specific to monocytes and macrophages, (Serotec, Oxford, United Kingdom), was diluted to 1:1 000. CD68-positive cells were readily detectable in the kidney tissue by light microscopy, and the number of CD68-positive cells was counted under ×400 magnification.

Western blot analysis

The kidney specimens were placed in RIPA buffer containing a protease inhibitor cocktail and homogenized. The tissue lysate was centrifuged, and the protein concentration of the resulting supernatant was determined using a protein assay kit (Bio-Rad Laboratories, Hercules, CA, USA). Samples containing 10 µg of total protein

were mixed with SDS loading buffer, boiled, electrophoresed in 10 % SDS-PAGE gels, and then transferred onto PVDF membranes. The membranes were then blocked with blocking buffer for 1 h at room temperature and incubated overnight at 4 °C with anti-oxLDL and anti-LOX-1 antibodies. After washing, the membranes were incubated with horseradish peroxidase-conjugated secondary antibodies. Immunoreactive protein detection was performed using an enhanced chemiluminescence detection system (PerkinElmer, Waltham, MA, USA).
